# Growth trajectory and miscarriage diagnosis threshold in early IVF pregnancy: multicenter study

**DOI:** 10.1002/uog.70349

**Published:** 2026-10-12

**Authors:** S. Murugesu, A. Richardson, A. Shearer, S. Sheth, D. Nikolaou, D. Balfoussia, M. Mittal, K. Linton‐Reid, E. O. Aboagye, S. Saso, S. Saravelos, T. Bourne

**Affiliations:** ^1^ Obstetrics and Gynaecology Department, Queen Charlotte's and Chelsea Hospital Imperial College Healthcare NHS Trust London UK; ^2^ Department of Metabolism, Digestion and Reproduction Imperial College London London UK; ^3^ CARE Fertility Nottingham UK; ^4^ Obstetrics and Gynaecology Department Chelsea and Westminster Hospital NHS Trust London UK; ^5^ Department of Cancer and Surgery Imperial College London London UK

**Keywords:** crown–rump length, early pregnancy, embryo transfer, *in‐vitro* fertilization, IVF, mean sac diameter, miscarriage

## Abstract

**Objectives:**

To characterize early‐pregnancy ultrasound findings in pregnancies conceived via *in‐vitro* fertilization (IVF) for which gestational age is known precisely based on the date of embryo transfer (ET), and to examine their association with first‐trimester pregnancy outcome.

**Methods:**

This was a retrospective multicenter cohort study of fresh‐ and frozen‐cycle IVF pregnancies from 14 centers across England between January 2022 and December 2024, with one center contributing additional data from January 2019 to December 2021. Patients with a confirmed intrauterine pregnancy at the first transvaginal ultrasound scan (≤ 9 + 6 weeks' gestation) after ET were included for analysis of pregnancy outcome. Data collected included the date of ET, embryo stage at transfer, early‐pregnancy ultrasound measurements, including crown–rump length (CRL) and mean sac diameter (MSD), and expected gestational age. The endpoint was viability at the 11–14‐week dating scan. CRL growth was modeled against gestational age using quadratic regression. The diagnostic performance of a range of gestational‐age cut‐offs for identifying miscarriage was evaluated using sensitivity, specificity and positive predictive value (PPV).

**Results:**

A total of 10 009 IVF pregnancies were included, of which 8223 (82.2%) were viable at 11–14 weeks and 1786 (17.8%) were non‐viable. A significant difference was found in the distribution of CRL measurements at the first ultrasound scan between pregnancies that were found to be viable and those that were non‐viable at the subsequent 11–14‐week scan (*P* < 0.001). When stratified by gestational age based on the date of ET, viable pregnancies had a higher median CRL than pregnancies of the same gestational age that subsequently ended in miscarriage, although there was a degree of overlap in the distribution of CRL values between the two groups. Furthermore, among pregnancies that were viable at 11–14 weeks, significant variability was seen in CRL and MSD measurements at each gestational age. The estimated mean CRL growth rate was 0.926 mm/day from when CRL was first measurable. The ultrasound finding of an empty gestational sac at or beyond 47 days' gestation based on the date of ET (*n* = 406) had a PPV of 100% and specificity of 100% (95% CI, 94.9–100%) for first‐trimester miscarriage.

**Conclusions:**

Early‐pregnancy ultrasound measurements in IVF pregnancy show wide physiological variability at any given gestational age, even when gestational age can be calculated precisely from the date of ET. An empty gestational sac at or beyond 47 days' gestation was associated with 100% PPV and 100% specificity for non‐viable pregnancy. Further external validation in independent cohorts is required to confirm this potential threshold for diagnosing miscarriage in IVF pregnancy. © 2026 The Author(s). *Ultrasound in Obstetrics & Gynecology* published by John Wiley & Sons Ltd on behalf of International Society of Ultrasound in Obstetrics and Gynecology.

## INTRODUCTION

To diagnose miscarriage, the clinician must have criteria that offer absolute accuracy. A misdiagnosis may lead to intervention that could terminate a healthy pregnancy. According to the guidelines of the UK National Institute for Health and Care Excellence (NICE), the diagnosis of miscarriage is based on a transvaginal ultrasound (TVS) examination demonstrating an empty gestational sac with a mean sac diameter (MSD) of ≥ 25 mm, or an embryo with a crown–rump length (CRL) of ≥ 7 mm and no heartbeat^1^, ^2^. These cut‐off values were derived from large studies and have achieved a positive predictive value (PPV) of 100% with a low risk of diagnostic error^3^.

Diagnostic guidelines advise that, when ultrasound findings suggest a pregnancy of unknown viability (PUV), a repeat scan should be performed at least 7 days later before making a diagnosis^1^. There is evidence to suggest that, in the case of an empty gestational sac with a MSD < 12 mm, a repeat scan at least 14 days later is required^4^. The calculation of gestational age from the last menstrual period (LMP) relies on patient recall, and ovulation does not reliably follow 2 weeks from the LMP, with a proportion of patients experiencing irregular cycles. Therefore, guidelines specify that LMP should not be used to determine a definitive miscarriage diagnosis^1^.

At present, there is no provision in the guidelines regarding a definitive miscarriage diagnosis for pregnancies conceived via *in‐vitro* fertilization (IVF), for which gestational age can be calculated precisely and reliably based on the date of embryo transfer (ET). Evaluating early‐pregnancy ultrasound measurements in the context of known gestational age may allow for more accurate prognostication at the first ultrasound scan. It is well documented that delays in receiving medical results heighten patient anxiety, with the level of distress shaped by both the waiting period and the potential significance of the outcome^5^, ^6^, ^7^.

We hypothesized that, in IVF pregnancies with a known ET date, there is a gestational‐age threshold at which the finding of an empty gestational sac will allow for earlier definitive diagnosis of miscarriage. This study aimed to characterize early‐pregnancy ultrasound findings, including growth measurements, in IVF pregnancies for which gestational age was known precisely based on the date of ET, and to examine whether these findings differed between pregnancies that remained viable and those that ended in miscarriage at the first‐trimester dating scan.

## METHODS

This was a retrospective multicenter cohort study of fresh‐ and frozen‐transfer IVF cycles from 14 IVF units in England, including two National Health Service (NHS) trusts in London (Imperial College Healthcare NHS Trust and Chelsea and Westminster Hospital NHS Foundation Trust) and 12 private IVF clinics (Care Fertility Group), between January 2022 and December 2024. We also included data collected at Hammersmith Hospital, Imperial College Healthcare NHS Trust, between January 2019 and December 2021. An analysis of the additional data collected from Hammersmith Hospital has been published previously^8^. Data collected as part of routine clinical care were extracted from the respective electronic health record system at each IVF unit. Pregnancies conceived by intracytoplasmic sperm injection were included.

In IVF pregnancy, gestational age at an early first‐trimester scan is calculated as follows:

Gestational age (days) = (date of initial scan – date of ET) (in days) + (age of embryo at transfer) (in days) + 14 days.

The addition of 14 days ensures that gestational age in IVF pregnancy is comparable with that in spontaneous pregnancy, which is dated from the LMP. In spontaneous pregnancy, gestational age at an early first‐trimester scan is calculated as follows:

Gestational age (days) = (date of initial scan – date of LMP).

At participating units, ET is performed at an embryo age of 3–6 days for fresh‐ and frozen‐transfer IVF cycles. Patients then undergo a pregnancy test to confirm pregnancy 2 weeks after ET. Those with a positive pregnancy test are booked in for their first ultrasound examination at ≤ 9 + 6 weeks' gestation. Possible diagnoses at this scan include PUV, viable intrauterine pregnancy and miscarriage. In our study, we included any patient that presented with a confirmed intrauterine pregnancy at this examination for analysis of pregnancy outcome.

Data collected included date of ET, embryo stage at transfer, CRL at the first early‐pregnancy scan (in the case of an empty gestational sac, the CRL was recorded as 0 mm), MSD at the first scan, and expected gestational age based on both the date of ET and embryo stage at transfer. The endpoint was viability at the first‐trimester dating scan, which was performed between 11 + 2 and 14 + 1 weeks. In cases of double ET, data were recorded at the level of the ET event; when two gestational sacs were identified at the 11–14‐week scan, outcome was recorded as two data points. We acknowledge that embryos within the same transfer are not statistically independent and share maternal and uterine factors. In cases in which a single gestational sac was observed, attribution of outcome to an individual embryo was not possible; however, the gestational age of the pregnancy was known and thus included in the analysis.

At the participating units, women whose pregnancy was confirmed as viable at the initial scan were advised to book antenatal care, while those diagnosed with miscarriage or PUV were referred to the local early pregnancy unit, where NICE guidelines were followed to determine follow‐up or diagnose miscarriage, as appropriate^1^. All women were followed up by the IVF units to record viability after the first trimester and the final outcome of the pregnancy.

TVS was performed using Voluson (GE Healthcare, Zipf, Austria) or Accuvix XG (Samsung Medison, Seoul, South Korea) machines. All machines used to make a final diagnosis in this study were less than 10 years old and subject to standard regular maintenance. MSD was calculated as the average of three orthogonal inner‐to‐inner gestational sac diameters, typically obtained in sagittal, transverse and coronal planes, in accordance with standard early‐pregnancy ultrasound practice. CRL was measured to the nearest 0.1 mm, with calipers placed at the outer side of the embryo's crown and rump (greatest length). The presence or absence of a visible heartbeat was recorded. Scans were performed by trained healthcare professionals, including sonographers, nurse practitioners and doctors with experience in early‐pregnancy ultrasound. Participating centers followed established clinical guidelines for early‐pregnancy assessment.

This study used anonymized data collected under routine clinical conditions. Ethical approval was granted by the Imperial College London Research Governance and Integrity Team (ICL RGIT), and the Health Research Authority (reference: 24/HRA/3688). This study was conducted and reported in accordance with the Standards for Reporting of Diagnostic Accuracy Studies (STARD) 2015 guideline^9^.

### Index test and reference standard

The index test was the first early‐pregnancy TVS examination performed at ≤ 9 + 6 weeks as part of routine clinical care. Operators performing the index test had access to relevant clinical information, including IVF treatment details and gestational age determined from the date of ET, reflecting standard clinical practice. The reference standard was viability at the 11–14‐week ultrasound scan, which was assessed prospectively during routine clinical follow‐up. No additional review or reinterpretation of ultrasound findings was performed for the purposes of this study.

### Statistical analysis

In this large multicenter dataset, a Kolmogorov–Smirnov test was applied to compare the CRL measurements of viable and non‐viable pregnancies in relation to expected gestational age based on the date of ET. The two cohorts were defined and separated by viability at the 11–14‐week ultrasound scan.

To characterize embryonic growth, quadratic polynomial regression modeling was used to fit CRL as a function of gestational age determined from the date of ET, measured in days. A quadratic model was selected to account for the curvilinear pattern of CRL growth observed in early pregnancy. The fitted regression curve was used to estimate the central growth trajectory among viable pregnancies, with 95% CI calculated to quantify the precision of the estimated curve. This model was used to estimate the mean CRL growth rate and to visualize natural variation in CRL measurements at each gestational age. Cases with missing outcome data were excluded prior to analysis. No imputation of missing ultrasound measurements or pregnancy outcomes was performed.

Sensitivity, specificity and PPV were used to evaluate the performance of various gestational age cut‐off values for identifying miscarriage at 11–14 weeks, for pregnancies in which the initial ultrasound scan showed an empty gestational sac. 95% CI were computed using Wilson's method. Statistical analysis was conducted using Python (version 3.13; https://www.python.org). *P* < 0.05 was considered statistically significant.

## RESULTS

This study included 10 009 IVF pregnancies, comprising 3782 (37.8%) fresh and 6227 (62.2%) frozen cycles. At the 11–14‐week ultrasound scan, 8223 (82.2%) pregnancies were confirmed as viable. Of the remaining 1786 (17.8%) non‐viable pregnancies, 1645 were confirmed non‐viable at the 11–14‐week scan; the other 141 cases had been diagnosed with miscarriage at the first early‐pregnancy ultrasound. When stratified by cycle type, the proportion of viable pregnancies was similar: 81.3% in the fresh cohort and 82.7% in the frozen cohort (*P* = 0.10). Pregnancy outcomes are summarized in Figure 1. The mean ± SD maternal and paternal ages at embryo transfer were 35.7 ± 4.4 years and 36.8 ± 5.7 years, respectively. The mean ± SD gestational age (based on the date of ET) at the first early‐pregnancy scan was 47.6 ± 4.7 days (*n* = 2446) at the NHS sites and 53.1 ± 4.8 days (*n* = 7563) at the private clinics (*P* < 0.01).

**Figure 1:**
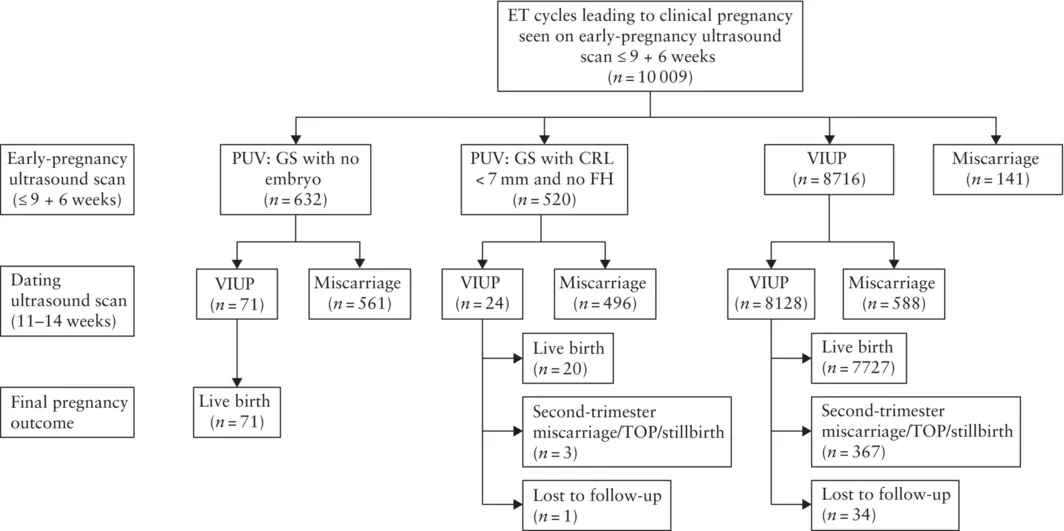
Flowchart summarizing outcome of 10 009 embryo transfer (ET) cycles leading to clinical pregnancy on early‐pregnancy ultrasound scan ≤ 9 + 6 weeks and with known outcome at end of first trimester. CRL, crown–rump length; FH, fetal heartbeat; GS, gestational sac; PUV, pregnancy of unknown viability; TOP, termination of pregnancy; VIUP, viable intrauterine pregnancy.

A significant difference was found in the distribution of CRL measurements at the first early‐pregnancy ultrasound scan between pregnancies that were found to be viable *vs* those that were non‐viable at the subsequent dating scan (*P* < 0.001). The smoothed kernel density estimate (KDE) plots in Figure S1 show CRL measurements for each gestational day between 6 + 0 and 9 + 0 weeks, stratified by viability at the 11–14‐week scan. The KDE plots depict a bell‐shaped curve of CRL measurements in pregnancies that went on to be viable, with a higher median CRL compared with the cohort of the same gestational age (based on the date of ET) that went on to miscarry, albeit with a degree of overlap.

CRL measurements by expected gestational age (based on the date of ET) in pregnancies confirmed as viable at the 11–14‐week dating scan are shown in Figure 2 and Table 1. A wide range of CRL values were recorded at each gestational age. A polynomial quadratic line with 95% CI was fitted to the data to model the rate of CRL increase at each day of gestation, chosen owing to the curvilinear trend of CRL measurements at early gestations, with the following equation:

**Figure 2:**
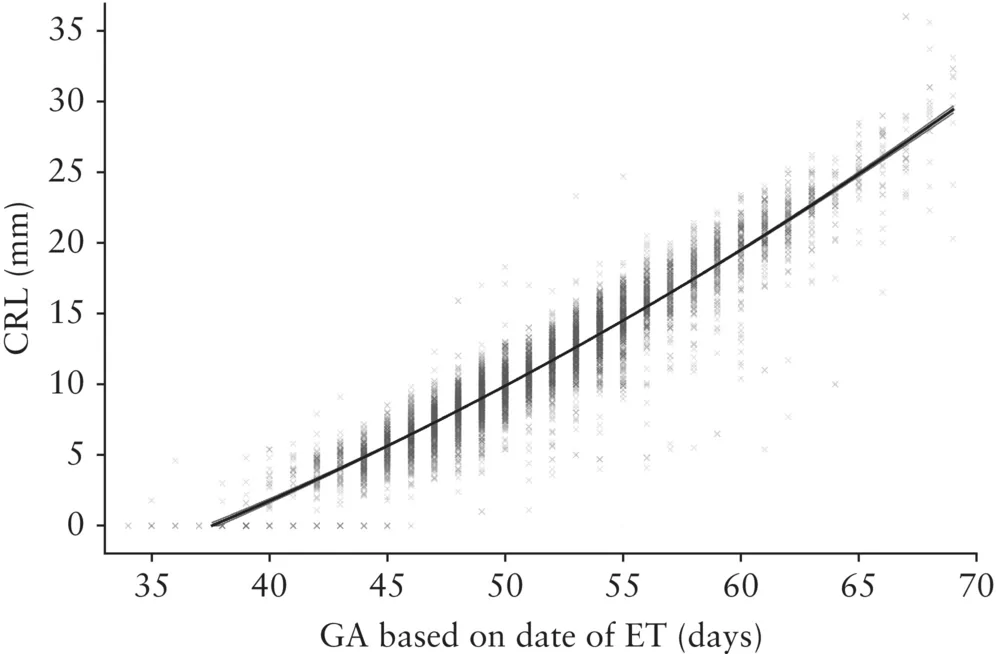
Relationship between crown–rump length (CRL) and expected gestational age (GA), based on date of embryo transfer (ET), at early‐pregnancy ultrasound scan (≤ 9 + 6 weeks) in pregnancies confirmed as viable at subsequent dating scan (11–14 weeks). Fitted polynomial quadratic curve (black line) with 95% CI (gray) for mean CRL is shown.

**Table 1:** Crown–rump length (CRL) measurements at early‐pregnancy ultrasound (US) scan (≤ 9 + 6 weeks) in 8223 pregnancies that were viable at dating scan (11–14 weeks), according to expected gestational age (GA) based on date of embryo transfer

Expected GA at US	Viable at dating scan (*n*)	Fetal pole absent (*n*)	Fetal pole present (*n*)	CRL measurement (mm)*, †
< 35 days	2	2	0	N/A
35 days	5	4	1	1.8
36 days	5	4	1	4.6
37 days	3	3	0	N/A
38 days	12	10	2	2.4 (1.7–3.0)
39 days	17	12	5	2.2 (1.6–4.8)
40 days	23	6	17	3.2 (1.2–5.4)
41 days	28	7	21	3.9 (2.0–5.8)
42 days	79	6	73	4.1 (1.1–7.9)
43 days	95	7	88	4.7 (1.5–9.1)
44 days	228	5	223	4.8 (2.1–7.2)
45 days	292	4	288	5.7 (1.8–8.5)
46 days	362	1	361	6.3 (2.0–9.8)
47 days	517	0	517	7.3 (3.3–12.3)
48 days	620	0	620	8.3 (2.4–15.9)
49 days	858	0	858	9.2 (1.0–17.0)
50 days	659	0	659	9.9 (5.4–18.3)
51 days	502	0	502	10.8 (1.1–17.0)
52 days	591	0	591	11.7 (3.6–16.6)
53 days	628	0	628	12.8 (5.0–23.3)
54 days	732	0	732	14.0 (4.0–18.5)
55 days	470	0	470	14.7 (9.0–24.7)
56 days	302	0	302	15.8 (4.1–20.5)
> 56 days‡	1193	0	1193	N/A

* Data are given as median or median (range).

† For cases in which fetal pole was present.

‡ CRL data beyond 56 days are presented as single aggregated category owing to progressively decreasing sample sizes at later GAs and because this range contains no cases with an empty gestational sac so has limited relevance to the study's diagnostic threshold for miscarriage. N/A, not applicable.

CRL (mm) = 0.00741*x* ^2^ + 0.1457*x* − 15.94, where *x* = gestational age based on date of ET (days).

The estimated mean CRL growth rate was 0.926 (95% CI, 0.919–0.933) mm/day from when CRL first exceeded 0 mm. When analyzed according to ET type, the mean CRL growth rate remained consistent: 0.934 (95% CI, 0.922–0.946) mm/day in fresh cycles and 0.924 (95% CI, 0.915–0.933) mm/day in frozen cycles. This difference was not statistically significant (ANCOVA interaction test, *P* = 0.197), indicating similar growth trajectories across both groups.

Of the 10 009 IVF pregnancies analyzed, 632 (6.3%) were classified as a PUV at the initial ultrasound scan, based on the finding of an intrauterine gestational sac without a visible embryo. This subgroup was analyzed separately, because current guidelines advise waiting a further 14 days before a miscarriage can be diagnosed definitively in these patients. MSD measurements by expected gestational age (based on the date of ET) in pregnancies with an empty gestational sac are shown in Figure 3, stratified by viability at the 11–14‐week scan. In this subgroup, there was a range of MSD measurements at each gestational age on the initial ultrasound scan among pregnancies that were viable at the subsequent dating scan. Within double‐embryo transfer cycles, no pregnancy presenting with empty gestational sacs resulted in a viable outcome. When an empty gestational sac was found at or beyond 47 days' gestation based on the date of ET (*n* = 406), all cases resulted in miscarriage (Figure 3).

**Figure 3:**
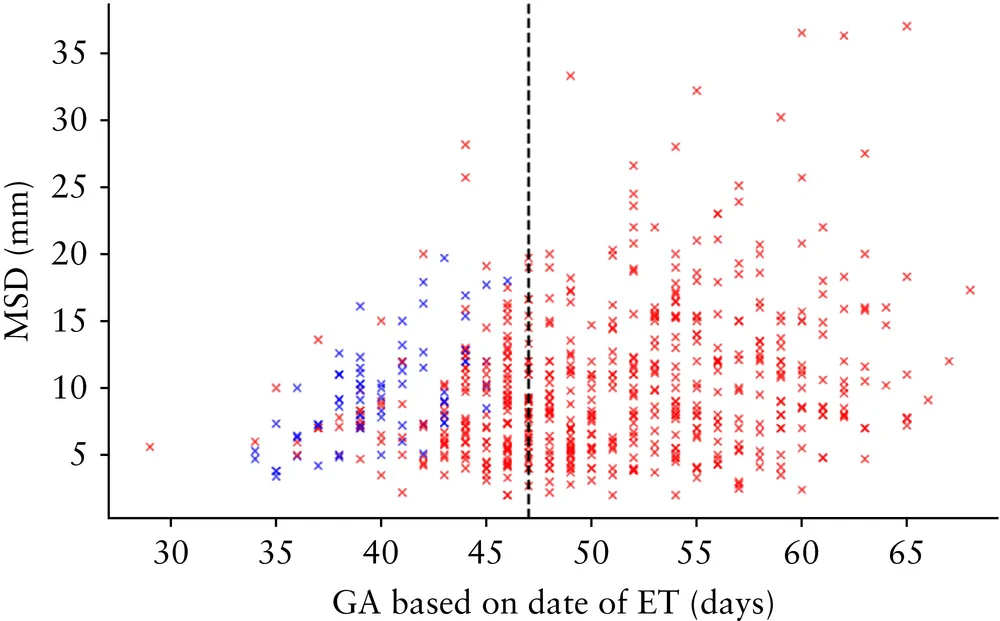
Scatter plot showing mean sac diameter (MSD) by expected gestational age (GA), based on date of embryo transfer (ET), at early‐pregnancy ultrasound scan (≤ 9 + 6 weeks) in pregnancies that presented with gestational sac with absent fetal pole. Data points are color‐coded according to whether pregnancy was viable (blue) or non‐viable (red) at subsequent dating scan (11–14 weeks). Dashed line indicates 47‐day GA threshold, beyond which all cases went on to be non‐viable.

Table 2 summarizes the diagnostic performance of various gestational age cut‐offs at the early‐pregnancy ultrasound scan for the identification of non‐viable pregnancy at 11–14 weeks, in cases in which the early‐pregnancy scan showed a gestational sac without a fetal pole (*n* = 632). All pregnancies with this ultrasound finding observed at or beyond 47 days' gestation had a PPV of 100% and specificity of 100% (95% CI, 94.9–100%) for miscarriage.

**Table 2:** Diagnostic performance of different cut‐off values of expected gestational age (GA), based on date of embryo transfer (ET), at early‐pregnancy ultrasound (US) scan (≤ 9 + 6 weeks), in cases in which initial US findings were gestational sac with absent fetal pole (*n* = 632), for prediction of non‐viable pregnancy at dating scan (11–14 weeks)

	First scan < threshold			First scan ≥ threshold					
Expected GA threshold	Total (*n*)	Viable at dating scan (TN) (*n*)	Non‐viable at dating scan (FN) (*n*)	Total (*n*)	Viable at dating scan (FP) (*n*)	Non‐viable at dating scan (TP) (*n*)	Sensitivity (95% CI) (%)	Specificity (95% CI) (%)	PPV (95% CI) (%)
35 days	4	2	2	628	69	559	99.6 (98.7–99.9)	2.8 (0.8–9.7)	89.0 (86.3–91.2)
36 days	9	6	3	623	65	558	99.5 (98.4–99.8)	8.5 (3.9–17.2)	89.6 (86.9–91.7)
37 days	15	10	5	617	61	556	99.1 (97.9–99.6)	14.1 (7.8–24.0)	90.1 (87.5–92.2)
38 days	22	13	9	610	58	552	98.4 (97.0–99.2)	18.3 (11.0–28.8)	90.5 (87.9–92.6)
39 days	38	23	15	594	48	546	97.3 (95.6–98.4)	32.4 (22.7–43.9)	91.9 (89.4–93.9)
40 days	58	35	23	574	36	538	95.9 (93.9–97.3)	49.3 (38.0–60.7)	93.7 (91.4–95.4)
41 days	73	41	32	559	30	529	94.3 (92.1–95.9)	57.7 (46.2–68.5)	94.6 (92.4–96.2)
42 days	86	48	38	546	23	523	93.2 (90.8–95.0)	67.6 (56.1–77.3)	95.8 (93.8–97.2)
43 days	100	54	46	532	17	515	91.8 (89.2–93.8)	76.1 (65.0–84.5)	96.8 (94.9–98.0)
44 days	122	61	61	510	10	500	89.1 (86.3–91.4)	85.9 (76.0–92.2)	98.0 (96.4–98.9)
45 days	153	66	87	479	5	474	84.5 (81.3–87.3)	93.0 (84.6–97.0)	99.0 (97.6–99.6)
46 days	181	70	111	451	1	450	80.2 (76.7–83.3)	98.6 (92.4–99.8)	99.8 (98.8–100)
47 days	226	71	155	406	0	406	72.4 (68.5–75.9)	100 (94.9–100)	100 (99.1–100)
48 days	264	71	193	368	0	368	65.6 (61.6–69.4)	100 (94.9–100)	100 (99.0–100)
49 days	297	71	226	335	0	335	59.7 (55.6–63.7)	100 (94.9–100)	100 (98.9–100)
50 days	335	71	264	297	0	297	52.9 (48.8–57.0)	100 (94.9–100)	100 (98.7–100)
51days	360	71	289	272	0	272	48.5 (44.4–52.6)	100 (94.9–100)	100 (98.6–100)
52 days	382	71	311	250	0	250	44.6 (40.5–48.7)	100 (94.9–100)	100 (98.5–100)
53 days	411	71	340	221	0	221	39.4 (35.4–43.5)	100 (94.9–100)	100 (98.3–100)
54 days	431	71	360	201	0	201	35.8 (32.0–39.9)	100 (94.9–100)	100 (98.1–100)
55 days	462	71	391	170	0	170	30.3 (26.6–34.2)	100 (94.9–100)	100 (97.8–100)
56 days	488	71	417	144	0	144	25.7 (22.2–29.4)	100 (94.9–100)	100 (97.4–100)

Widening 95% CI for positive predictive value (PPV) beyond 47 days' gestation reflects sample size effect. FN, false negative; FP, false positive; TN, true negative; TP, true positive.

## DISCUSSION

This large multicenter study of 10 009 IVF pregnancies validates and strengthens a previously proposed diagnostic threshold for miscarriage, narrowing CIs and improving generalizability^8^. Our findings suggest that, when gestational age can be determined precisely based on the date of ET, miscarriage can be diagnosed with 100% specificity at or beyond 47 days' gestation if the ultrasound scan shows an empty gestational sac. This supports a potential revision to current diagnostic criteria for miscarriage in IVF pregnancies.

This study contributes detailed insight into CRL growth trajectories in viable IVF pregnancies. Several prior studies have established CRL‐based reference charts to estimate gestational age, including the Robinson and Hadlock curves^10^, ^11^. However, the Robinson curve was derived from a small population using transabdominal sonography, and the Hadlock curve was generated using early TVS equipment; both used mixed populations dated by LMP and carry intrinsic limitations in early gestation.

Delpachitra *et al*.^12^ addressed this by using fresh‐cycle IVF pregnancies (*n* = 1268) with known ET date to produce the ‘Monash Chart’, demonstrating systematic discrepancies when IVF pregnancies were assessed using traditional LMP‐based nomograms. Our estimated growth rate (0.926 mm/day) offers updated growth benchmarks for mixed fresh‐ and frozen‐cycle IVF pregnancies in a larger, more heterogeneous, multicenter cohort.

While the overall growth trend was consistent, there was a range of CRL measurements at each timepoint among pregnancies that went on to be viable; for example, at 48 days, the CRL range was 2.4–15.9 mm. This variation probably reflects both biological and technical factors, including intrinsic embryo quality/genetic factors^13^, ^14^, implantation dynamics^15^ and interoperator variability^16^. For instance, Wilcox *et al*.^15^ found that later implantation timing was associated with increased risk of early pregnancy loss in spontaneous pregnancy. Notably, our analysis demonstrated no significant difference in growth rate or miscarriage risk between fresh and frozen IVF cycles. The variation in viable early‐pregnancy CRL measurements highlights the importance of using robust CRL thresholds stipulated by national guidelines for the diagnosis of miscarriage in the presence of an embryo.

The finding that no pregnancy with an empty gestational sac ≥ 47 days progressed to viability builds upon our previous single‐center study, which first proposed the 47‐day threshold^8^. That study was limited by a small sample size around the critical threshold and a wider confidence interval for specificity (95% CI, 90.1–100%). The present multicenter study validates the threshold in a substantially larger cohort, with a PPV of 100% and a narrower CI for specificity (95% CI, 94.9–100%), improving generalizability and clinical applicability. Notably, this cohort overlaps with our earlier single‐center study rather than representing a fully independent replication; validation in a fully separate cohort remains an important next step.

Timing of the first early‐pregnancy ultrasound scan varied across units, with the two NHS units conducting the first scan earlier on average compared with the 12 private‐sector fertility units. This explains why the number of PUV cases increased only 4‐fold (157 to 632) compared with our previous study conducted in a single NHS unit, despite an 8‐fold increase in the number of ET cycles analyzed (1161 to 10 009)^8^. In a study on the optimal timing of an ultrasound scan to assess the location and viability of an early pregnancy, Bottomley *et al*.^17^ found that the most common TVS findings were pregnancy of unknown location (PUL) before 35 days, PUV from 35 to 41 days and viable intrauterine pregnancy from 42 days onwards. These findings are consistent with those of our study, demonstrating how a protocol for later gestational age at first ultrasound scan leads to a lower number of PUV cases. Importantly, our study demonstrates that it is possible to have an empty gestational sac at ≤ 46 days' gestation and still have a viable pregnancy.

Detti *et al*.^18^ developed a predictive nomogram for miscarriage incorporating CRL, gestational sac diameter and fetal heart rate across weekly gestational windows. Our findings, using the precision afforded by ET timing, expand this approach by offering a single‐day diagnostic threshold for miscarriage with 100% specificity and 100% PPV within a large IVF cohort. Dooley *et al*.^19^ also determined that gestational age could inform clinical decision‐making in women with a PUL following IVF, as no viable intrauterine pregnancy was found in their cohort beyond 5 weeks' gestation with a TVS finding of PUL.

Our results suggest that an empty gestational sac in an IVF pregnancy may permit an earlier diagnosis of miscarriage than current NICE guidance allows^1^. Earlier definitive diagnosis of miscarriage could help to alleviate the psychological burden of uncertainty for patients, reduce unnecessary follow‐up scans and permit cessation of IVF medication. As with any diagnostic criteria for miscarriage, women who meet the criteria could still choose to undergo conservative management with repeat scans, or to pursue definitive medical or surgical management, as per patient preference. Further validation and evaluation are needed before such an addition to miscarriage diagnosis is adopted in clinical practice.

A major strength of this study is its large multicenter design involving over 10 000 IVF cycles, with 632 cases of an empty gestational sac at the first early‐pregnancy ultrasound scan. ET‐based dating allows for precise calculation of gestational age, removing one of the major sources of uncertainty in early‐pregnancy ultrasound. However, several limitations should be acknowledged. Although the data were collected prospectively as part of routine clinical care, this analysis was retrospective and dependent on measurements recorded during clinical visits. Additionally, while scan protocols were broadly standardized across sites and adhered to NICE guidelines^1^, variations in measurement technique between operators may have contributed to the observed variability in early‐pregnancy measurements. However, this reflects the real‐world multicenter nature of the dataset and enhances the external validity of the findings.

Pregnancies with an embryo measuring < 7 mm without a detectable fetal heartbeat represent another clinically important subgroup. However, as current guidelines require a shorter interval for follow‐up in this group compared to those with an empty gestational sac, our analysis prioritized the latter cohort. Future studies could evaluate whether discrepancies between expected gestational age based on the date of ET and observed CRL measurements provide additional diagnostic value. While this study characterized CRL growth trajectories in viable pregnancies, we did not evaluate whether early CRL measurements falling into lower percentiles are associated with subsequent adverse outcome; this represents an important area for future research.

In conclusion, this large multicenter study suggests that an empty gestational sac ≥ 47 days' gestation, calculated based on the date of ET, may represent a diagnostic threshold for non‐viability in IVF pregnancy. However, given the retrospective design and wide CIs, external validation in independent cohorts is required before clinical implementation can be considered. Detailed analysis of CRL growth in viable IVF pregnancies confirmed consistent growth trajectories while highlighting significant individual variability. Variation in the size of embryos and gestational sacs at each gestational age among subsequently viable pregnancies demonstrates that assumptions about viability cannot be made based on size and gestational age, unless they meet specific validated diagnostic thresholds to confirm miscarriage.

## Supporting information


**Figure S1** Smoothed kernel density estimate plots showing distribution of crown–rump length (CRL) measurements by expected gestational age (GA), based on date of embryo transfer, at early‐pregnancy ultrasound scan (≤ 9 + 6 weeks), in pregnancies that went on to be viable (blue) or non‐viable (red) at subsequent dating scan (11–14 weeks). Median with ± 1 SD are shown as dotted lines for visual reference.

## Data Availability

The data that support the findings of this study are available on request from the corresponding author. The data are not publicly available due to privacy or ethical restrictions.
